# Supplementary material for: Genomic regression analysis of coordinated expression
Source: Nat Commun. 2017 Dec 19;8:2187. doi: 10.1038/s41467-017-02181-0 (PMC5736603; doi:10.1038/s41467-017-02181-0)
Supplement: Supplementary file 1 — Supplementary Information [file 41467_2017_2181_MOESM1_ESM.pdf]

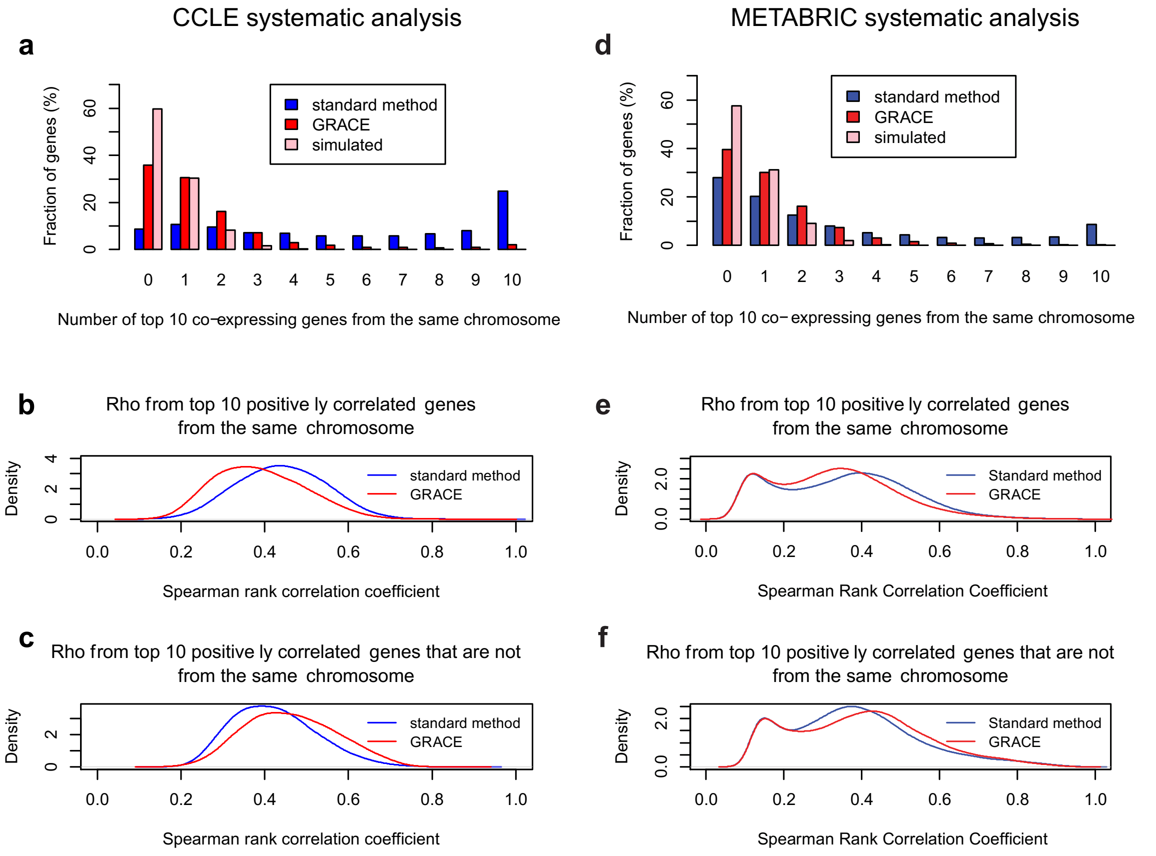

Supplementary Figure 1. GRACE corrects for correlation bias from copy number variation in CCLE cell line data and METABRIC discovery set data

a, Relative frequency distribution of chromosomal neighbors in top 10 co-expressing genes for all genes based on CCLE cell line data. **b**, **c**, Kernel density estimation plots that visualize the distribution of pooled Spearman rank correlation coefficients for top 10 co-expressing genes from the same chromosome (**b**) or not from the same chromosome (**c**) based on CCLE cell line data. **d**, Relative frequency distribution of chromosomal neighbors in top 10 co-expressing genes for all genes based on METABRIC discovery set data. **e**, **f**, Kernel density estimation plots that visualize the distribution of pooled Spearman rank correlation coefficients for top 10 co-expressing genes from the same chromosome (**e**) or not from the same chromosome (**f**) based on METABRIC discovery set data.

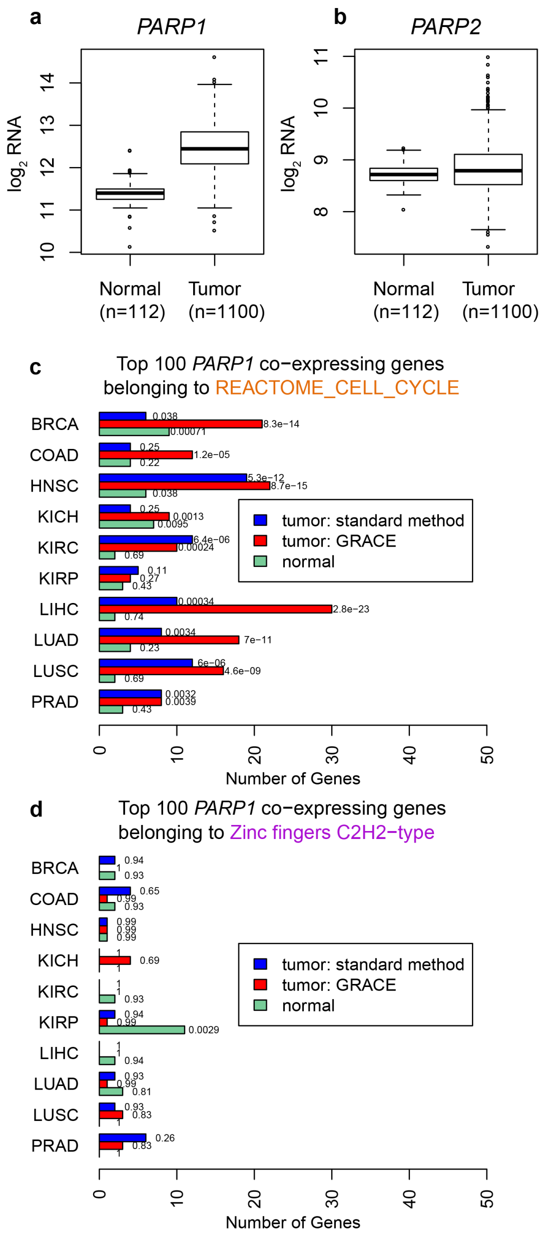

Supplementary Figure 2. *PARP1* levels and enrichment in different gene sets

a, **b**, Levels of *PARP1* (**a**) and *PARP2* (**b**) in normal and tumor samples. *PARP1* but not *PARP2* is upregulated in the tumor samples compared to the normal samples. In the boxplot, the lower whisker extends from the lower quartile to the lowest smaller value within 1.5 inter-quartile-range (IQR) whereas the upper whisker extends from the upper quartile to the highest larger value within 1.5 IQR. **c**, **d**, Enrichment of gene set “REACTOME_CELL_CYCLE” (**c**) or “Zinc fingers, C2H2-type” (**d**) in the top 100 *PARP1* co-expressing genes by standard method or GRACE in tumor or normal tissues from different TCGA cohorts. Similar to *PARP2*, PARP1 co-expressing genes in tumor tissues are enriched in cell cycle genes, but unlike *PARP1*, *PARP2* co-expressing genes from normal samples are not enriched in genes encoding C2H2-type zinc finger proteins.

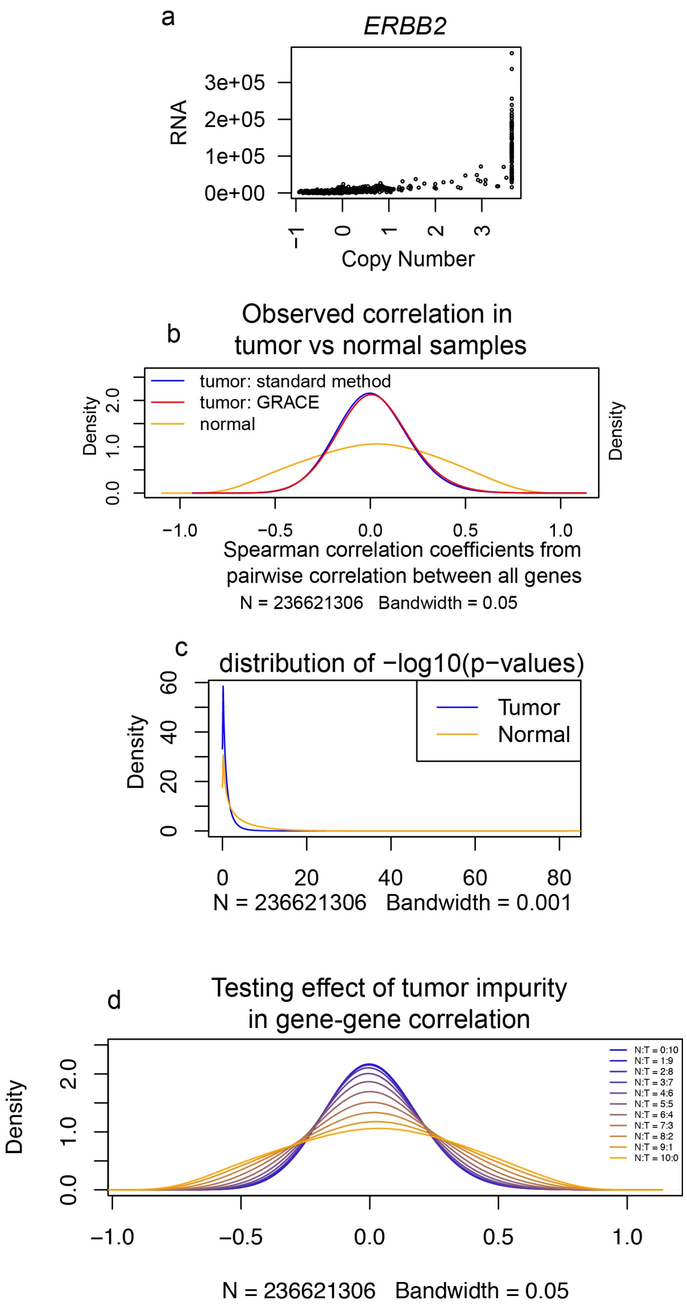

Supplementary Figure 3. Limitations of GRACE

a, Copy number versus RNA levels of ERBB2 from tumor samples. **b**, Kernel density estimation plots that visualize the distribution of pooled Spearman rank correlation coefficients for pairwise correlation from all the genes using tumor samples (by standard method or GRACE) or normal samples. Analyses are based on TCGA BRCA data. **c**, The number of significant pairwise gene correlations calculated from normal tissue data is higher than that from the tumor tissue data. **d**, Distribution of correlation coefficients from synthetic samples that had matched tumor and normal sample expression data mixed together at different ratios. All analyses are based on TCGA BRCA data.

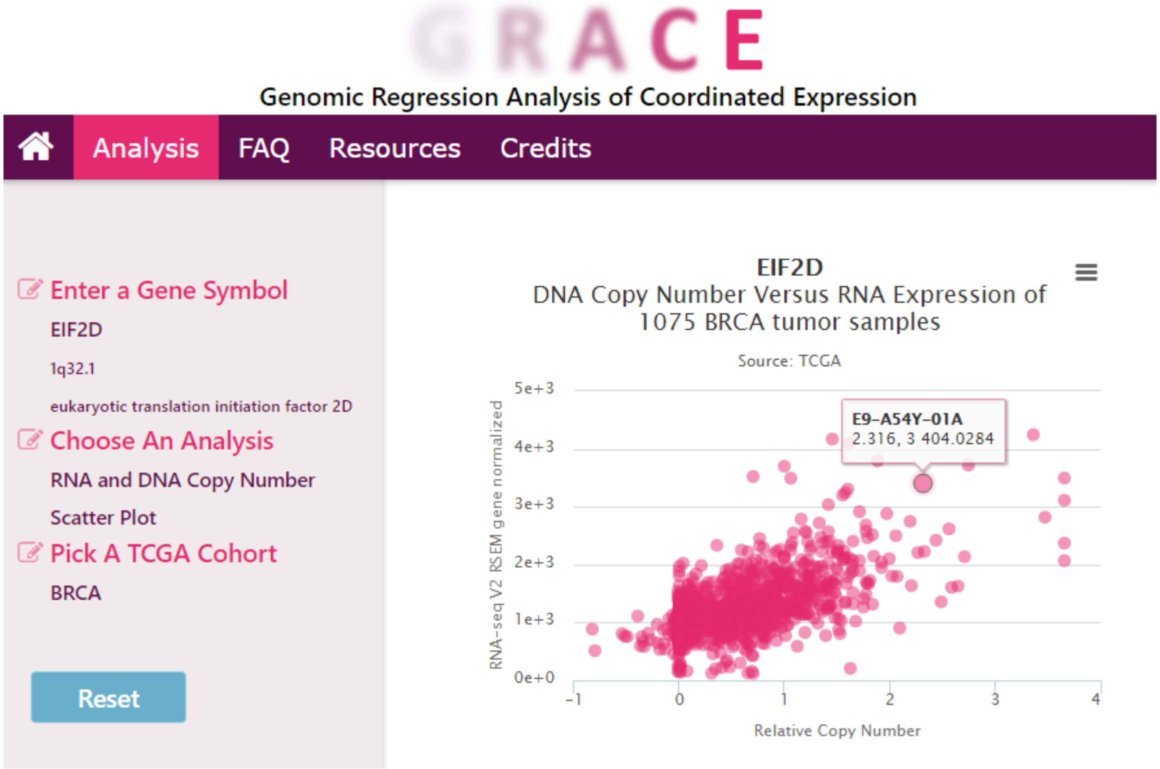

Supplementary Figure 4. RNA and DNA copy number scatter plot in web database GRACE

In the analysis page of the web database GRACE, upon entry of gene name, selection of analysis, and cohort, a scatter plot of RNA and relative DNA copy number will be generated. Users may hover the pointer over data points to examine the exact RNA and DNA copy number values as well as the TCGA label for the sample.

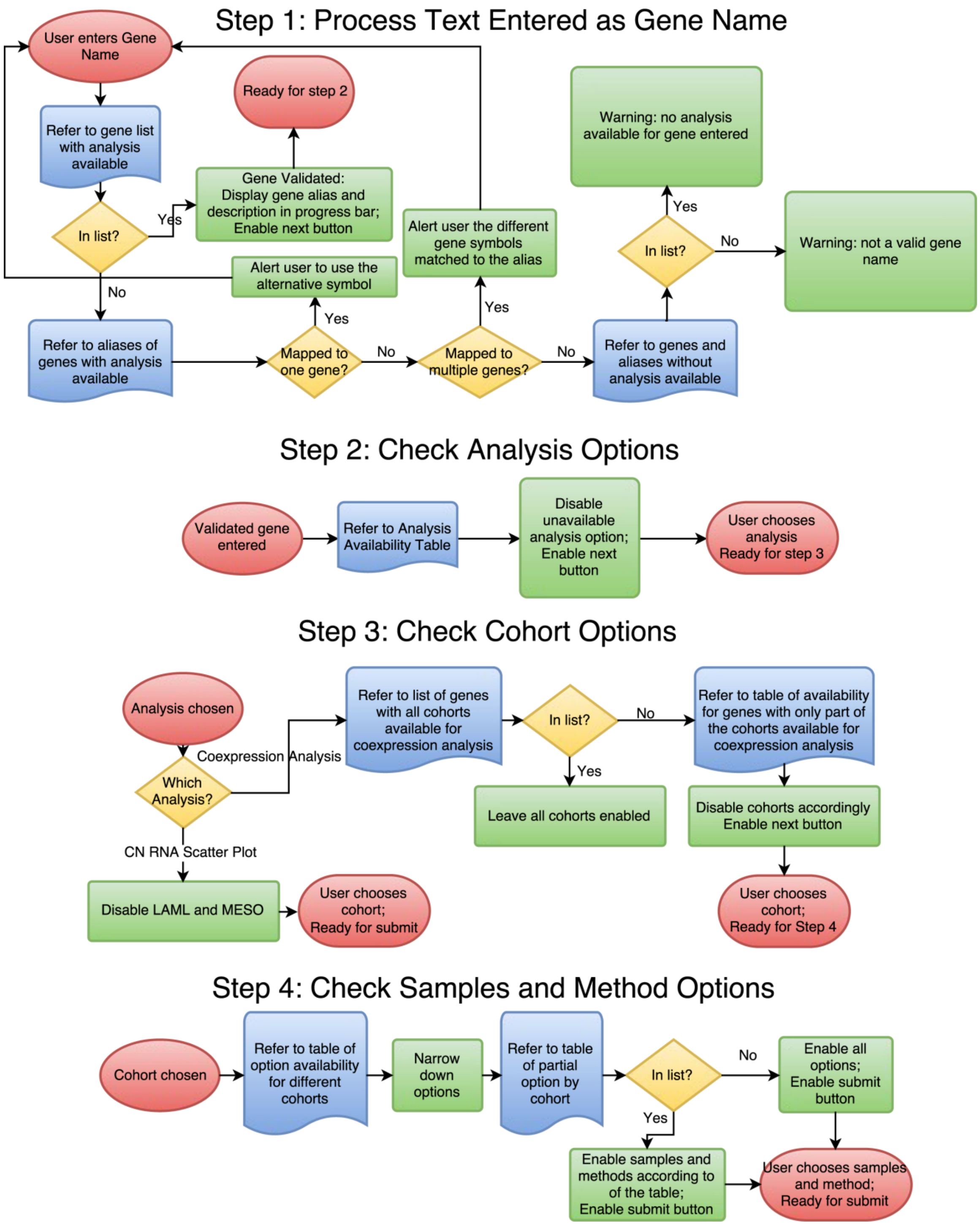

Supplementary Figure 5. Flowchart for stepwise analysis configuration and relational database design for GRACE web database

The stepwise analysis options are configured in a way that users will be alerted to wrong inputs or genes without data available for analysis in the first step; and in subsequent steps, only options with data available for analysis will be enabled.

Supplementary Note

In this study, we developed a method to adjust the effect of copy number alterations on gene expression in co-expression analysis of genes, together with a web-portal. We proposed an approximation method to improve the computation efficiency of the inference on large data sets with thousands of genes. Our method used a regression model to fit gene expression using copy number as predictors, and calculated the residuals, which is the gene expression values after adjusting for the copy number values. Our model computes the pairwise correlation of each pair of genes, taking account of the influence of the copy numbers. In this supplementary note, we use simulation studies to demonstrate that the calculated pairwise correlations approximate the true partial correlations in practice if the gene expression data follows Gaussian graphical models (GGMs).

Let $\boldsymbol{x}_i = (x_{i1} \quad \cdots \quad x_{ij} \quad \cdots \quad x_{ip})$ denote the copy number values of genes $1, \quad \cdots, \quad p$ in sample i. According to Gaussian graphical models (GGM), which are commonly used to infer the gene regulatory network, we simulated the gene expression levels $\boldsymbol{y}_i = (y_{i1} \quad \cdots \quad y_{ij} \quad \cdots \quad y_{ip})$ for sample i from a multivariate normal distribution $\boldsymbol{y}_i \sim \mathrm{MN}(\boldsymbol{b}_0 + \boldsymbol{b}_1 \circ \boldsymbol{x}_i, \boldsymbol{\Sigma} + \varepsilon^2 \boldsymbol{I})$, where $\circ$ denotes the Hadamard product (i.e. entry-wise product) and $\boldsymbol{I}$ denotes the p-by-p identity matrix. For the copy numbers $(\boldsymbol{x}_1 \quad \cdots \quad \boldsymbol{x}_n)$, we borrowed the real data from TCGA BRCA cohort, which contains copy numbers of $18{,}680$ genes for $n = 1{,}075$ samples. We selected $p = 1000$ genes at random. For the gene-specific coefficients $\boldsymbol{b}_0 = (b_{01} \quad \cdots \quad b_{0j} \quad \cdots \quad b_{0p})$ and $\boldsymbol{b}_1 = (b_{11} \quad \cdots \quad b_{1j} \quad \cdots \quad b_{1p})$, we drew each element from $b_{0j} \sim \mathrm{N}(0, 1)$ and $b_{1j} \sim \mathrm{N}(1, 0.5)$, respectively, where the latter imitates a positive relationship between gene copy number and expression. For the covariance matrix $\boldsymbol{\Sigma}$, we generated its concentration matrix $\boldsymbol{\Omega} = \boldsymbol{\Sigma}^{-1}$ as described below [1]). The initial p-by-p matrix $\boldsymbol{\Omega}$ was created by setting $\omega_{jh} = \begin{cases} 1, \ j = h \\ 0, \ j \neq h, j \nsim h \\ 0.5\mathrm{U}(-1, -0.5) + 0.5\mathrm{U}(0.5, 1), \ j \neq h, j \sim h \end{cases}$, where $j \sim h$ indicates that there is an edge between gene j and h, $j \nsim h$ means otherwise. Then, the non-zero elements in $\boldsymbol{\Omega}$ were rescaled to assure positive definiteness. Specifically, for each row, we first summed the absolute values of the off-diagonal elements, and then divided each off-diagonal entry by 1.5 fold of the sum. We then averaged this rescaled matrix with its transpose to ensure symmetry. The inverse of the final matrix was denoted by $\boldsymbol{A} = \boldsymbol{\Omega}^{-1}$. Therefore, the covariance matrix $\boldsymbol{\Sigma}$ was determined by $\sigma_{jh} = a_{jh} / \sqrt{a_{jj} a_{hh}}$. For the network structure, we assumed that it was composed of disjointed modules as many real biological networks exhibit such a feature. Each of $K = 5$ modules had 200 vertices. For the same network, all modules followed the same network model (but are not necessarily the same setting). The three major network models that we considered are: autoregressive (AR) model, Barabási-Albert (BA) model, and Erdős-Rényi (ER) model. Specifically, for the AR model, we took a ring with all vertices and connected each vertex to its nearest 4 neighbors; for the BA model, we set the power parameter to 2, as many real biological networks have a scale-free degree distribution with an estimated power parameter $2 \sim 3$ [2]; for the ER model, we randomly connected each pair of vertices with probability 1%.

To assess the accuracy of GRACE under different noise levels, $\varepsilon = 0$, 0.1, 0.5, 1 and different network models, there were $4 \times 3 = 12$ group of synthetic datasets generated. For each group, 100 datasets were independently simulated. To quantify the performance, we used the root-mean-square error (RMSE) to measure the differences between the true correlation matrix $\boldsymbol{P} = \left(\mathrm{diag}(\boldsymbol{\Sigma})\right)^{-1/2} \boldsymbol{\Sigma} \left(\mathrm{diag}(\boldsymbol{\Sigma})\right)^{-1/2}$ and the estimated one $\widehat{\boldsymbol{P}}$ by GRACE, calculated by $\mathrm{RMSE} = \frac{\sum_{j<h}\left(\rho_{jh} - \hat{\rho}_{jh}\right)^2}{p(p-1)/2}$. The boxplot of RMSEs under different settings are displayed in Supplementary Figure 6. It shows that the estimated correlations by GRACE are good approximations of their true values, especially when the noise level is at a low level. Among the three network models, there is not much difference when $\varepsilon < 0.5$. However, if the noise level becomes stronger, the AR and BA models outperform the ER model. We also plotted the scatter points of the true and estimated correlation matrix for one of the synthetic datasets in the group, for which $\varepsilon = 0.1$ and the network model is ER. As shown in Supplementary Figure 7, again, the estimated correlations by GRACE are good approximations of the truth with $\mathrm{RMSE} = 0.00093$. In summary, GRACE provides a good approximation of the partial correlation, and it greatly improves the computation efficiency. Furthermore, Supplementary Figure 8 shows the result when we directly calculated the correlations of expression levels between each pair of genes, without considering the effect of copy number values on gene expression levels. As we can see, it fails to recover the truth and results in a number of false positives, so it is important to adjust the copy number in the co-expression analysis.

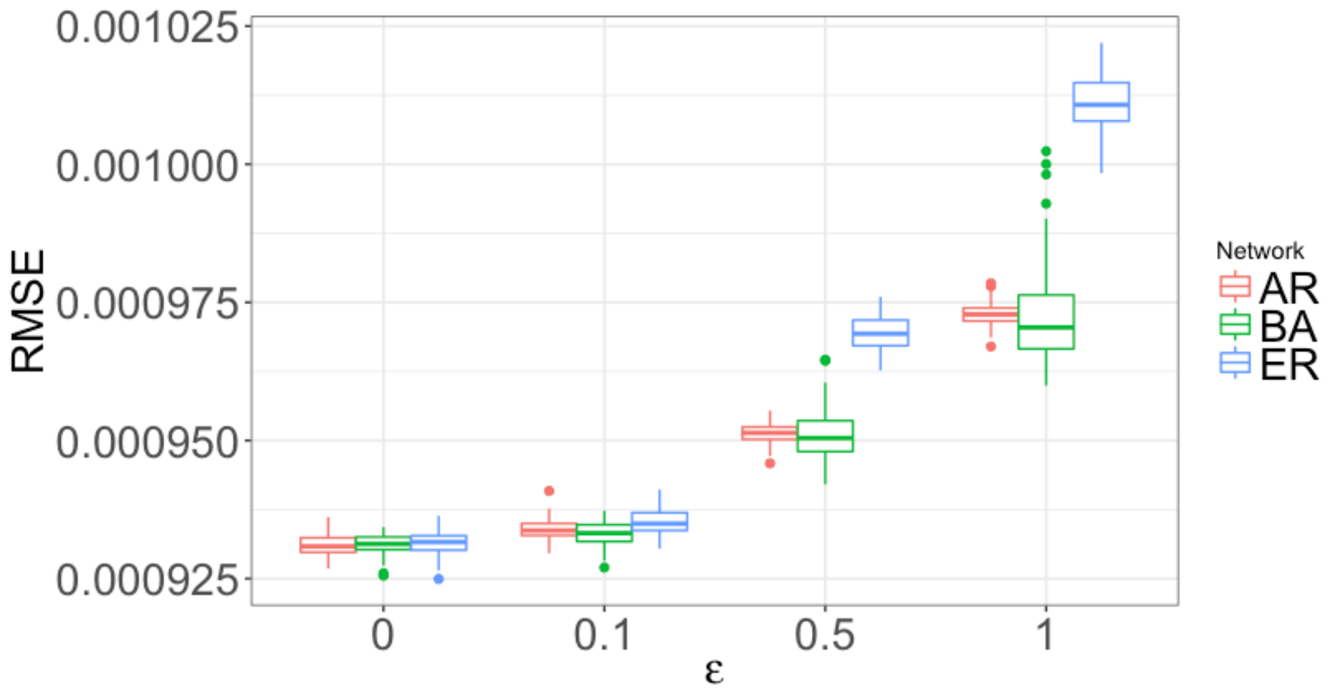

Supplementary Figure 6. The boxplots of RMSEs under different network models (AR, BA, and ER) and different noise levels ε.

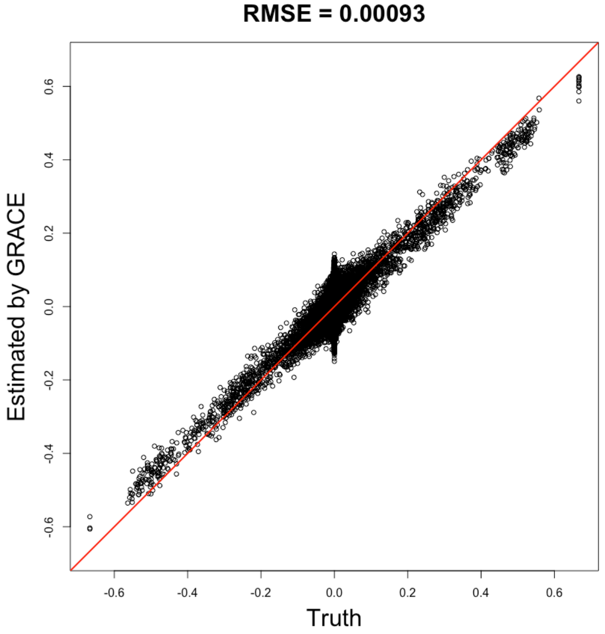

Supplementary Figure 7. The scatter plot of the upper-triangle entries of the true correlation matrix $\left(\mathrm{diag}(\boldsymbol{\Sigma})\right)^{-1/2}\boldsymbol{\Sigma}\left(\mathrm{diag}(\boldsymbol{\Sigma})\right)^{-1/2}$ and of the estimated correlation matrix by GRACE.

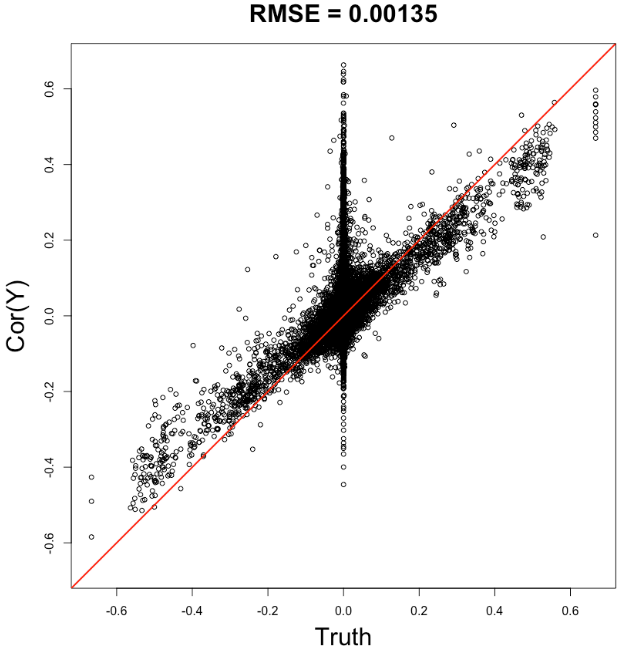

Supplementary Figure 8. The scatter plot of the upper-triangle entries of the true correlation matrix $\left(\mathrm{diag}(\boldsymbol{\Sigma})\right)^{-1/2}\boldsymbol{\Sigma}\left(\mathrm{diag}(\boldsymbol{\Sigma})\right)^{-1/2}$and of the correlation matrix of gene expression levels data $\mathrm{corr}(y_1 \quad \cdots \quad y_p)$.

In addition, we provide the justification of our statistical method below.

The residual for gene j is defined as $r_j = Y_j - \beta_j X_j = \alpha_{jh} Y_h + \varepsilon_j$.

Assuming X_j and Y_h are independent for all $j \neq h$, the covariance between two residuals r_j and r_h is $\mathrm{Cov}(r_j, r_h) = \alpha_{jh}\alpha_{hj}\mathrm{Cov}(Y_j, Y_h)$.

Plugging the regression model into Y_h, we obtain

$$\mathrm{Cov}(r_j, r_h) = \alpha_{jh}\alpha_{hj}\mathrm{Cov}(Y_j, \alpha_{hj} Y_j) = \alpha_{jh}\alpha_{hj}^2$$

Similarly, we have $\mathrm{Cov}(r_h, r_j) = \alpha_{jh}^2\alpha_{hj}$, which implies $\alpha_{hj} = \alpha_{jh}$. Then, the correlation between r_j and r_h can be written as

$$\rho_{jh} = \frac{\alpha_{jh}^3}{\sqrt{(\alpha_{jh}^2 + \sigma_j{}^2)(\alpha_{jh}^2 + \sigma_h{}^2)}}$$

From our experience, our regression model yields a high coefficient of determination (i.e. R^2), which provides an upper bound for $\sigma_j^2 \leq (1 - R_j)^2 \approx 0$ and $\sigma_h^2 \leq (1 - R_h)^2 \approx 0$. Hence, ρ_{jh} can be considered as a close approximation of α_{jh}. Also, in a situation with low R_j^2 and R_h^2, the high correlation can be used as an indication for high value of $\alpha_{jh} = \alpha_{hj}$. As a result, the computation can be simplified to a calculation of the correlation of the residual, ρ_{jh}, instead of the α_{jh}.

In summary, our GRACE model is able to recover the true covariance structure of genes, as well as its corresponding precision matrix, which indicates co-expressed gene sets. This simulation study also demonstrates the power of GRACE under different noise levels and major network models.

Reference:

1. Peng, J., Wang, P., Zhou, N., & Zhu, J. (2009). "Partial correlation estimation by joint sparse regression models." *Journal of the American Statistical Association*, 104(486): 735-746 DOI:
2. Newman, M. E. (2003). "The structure and function of complex networks." *SIAM Review*, 45(2), 167-256 DOI:
