# Supplementary material for: Genomic regression analysis of coordinated expression
Source: Nat Commun. 2017 Dec 19;8:2187. doi: 10.1038/s41467-017-02181-0 (PMC5736603; doi:10.1038/s41467-017-02181-0)
Supplement: Supplementary file 3 — Description of Additional Supplementary Files [file 41467_2017_2181_MOESM3_ESM.pdf]

Description of Additional Supplementary Files

File Name: Supplementary Data 1
Description: Genes with different co-expressing genes in tumor vs normal tissue.

File Name: Supplementary Data 2
Description: Number of genes whose transcription levels are significantly correlated with orthogonl molecular features by RNA or residuals.
